# Allosteric Inhibition of P-Glycoprotein-Mediated Efflux by DMH1

**DOI:** 10.3390/biomedicines13081798

**Published:** 2025-07-23

**Authors:** Zhijun Wang, Chen Xie, Maggie Chou, Jijun Hao

**Affiliations:** 1 Department of Clinical Pharmacy Practice, School of Pharmacy & Pharmaceutical Sciences, University of California, Irvine, CA 92697, USA; 2 College of Veterinary Medicine, Western University of Health Sciences, Pomona, CA 91766, USA

**Keywords:** multidrug resistance, P-glycoprotein, DMH1, drug efflux, ATPase, allosteric

## Abstract

**Background/Objectives:** P-glycoprotein (P-gp), an ATP-binding cassette (ABC) transporter, plays a key role in multidrug resistance by actively exporting chemotherapeutic agents and xenobiotics from cells. Overexpression of P-gp significantly reduces intracellular drug accumulation and compromises treatment efficacy. Despite extensive research, clinically approved P-gp inhibitors remain elusive due to toxicity, poor specificity, and limited efficacy. This study investigates DMH1, a selective type I BMP receptor inhibitor, as a novel P-gp inhibitor. **Methods:** DMH1 cytotoxicity was assessed in P-gp-overexpressing (PC3-TxR, K562/Dox) and P-gp-deficient (PC3) cell lines using MTT assays. P-gp inhibition was evaluated using calcein AM retention and daunorubicin (DNR) accumulation assays. Kinetic analysis determined DMH1’s effect on P-gp-mediated transport (Vmax and Km). ATPase activity assays were performed to assess DMH1’s impact on ATP hydrolysis. Preliminary molecular docking (CB-Dock2) was used to predict DMH1’s binding site on the human P-gp structure (PDB ID: 6QEX). **Results:** DMH1 showed no cytotoxicity in P-gp-overexpressing or deficient cells. It significantly enhanced intracellular accumulation of Calcein AM and DNR, indicating effective inhibition of P-gp function. Kinetic data revealed that DMH1 reduced Vmax without affecting Km, consistent with noncompetitive, allosteric inhibition. DMH1 also inhibited ATPase activity in a dose-dependent manner. Docking analysis suggested DMH1 may bind to an allosteric site in the transmembrane domain, potentially stabilizing the inward-facing conformation. **Conclusions:** DMH1 is a promising noncompetitive, allosteric P-gp inhibitor that enhances intracellular drug retention without cytotoxicity, supporting its potential as a lead compound to overcome multidrug resistance and improve chemotherapeutic efficacy.

## 1. Introduction

P-glycoprotein (P-gp), also referred to as multidrug resistance protein 1 (MDR1), is a membrane protein involved in the body’s defense by actively exporting harmful substances out of cells. It belongs to the ATP-binding cassette (ABC) superfamily, utilizing energy from ATP hydrolysis to actively transport substances across cell membranes [1]. P-gp consists of two transmembrane domains (TMDs), which recognize and bind diverse substrate molecules, and two cytoplasmic nucleotide-binding domains (NBDs), which are structurally responsible for ATPase activity and mediate substrate efflux in an ATP-dependent manner [2,3,4]. P-gp is expressed in many normal tissues, such as hepatocytes in the liver, epithelial cells of proximal tubules in the kidneys, epithelial cells of the intestine, capillary endothelial cells in the brain, and placental epithelial cells, where P-gp protects the body from toxic foreign substances by eliminating them into bile, urine, and the intestinal lumen [5,6,7].

Nevertheless, overexpression of P-gp is not always beneficial. In cancer therapy, P-gp overexpression in tumor cells contributes to multidrug resistance by actively expelling therapeutic agents, thereby reducing their intracellular concentrations and diminishing treatment efficacy [4,8]. Inhibiting P-gp activity can enhance intracellular drug accumulation, potentially overcoming resistance and improving therapeutic outcomes. As a result, significant efforts have been made toward developing P-gp inhibitors to overcome drug resistance in cancer treatment. To date, numerous P-gp inhibitors, including verapamil, cyclosporine A, laniquidar, and valspodar, as well as natural products and chemically modified natural compounds, have been reported to restore the sensitization of cancer cells to the chemotherapeutic agents [1,9,10,11,12]. However, none of these inhibitors have been proven to be clinically successful due to limitations such as poor selectivity, toxicity, and insufficient efficacy in clinical settings [13,14,15]. Therefore, identifying new P-gp inhibitors with improved safety profiles, higher specificity, and greater potency is highly sought after.

DMH1 is a selective inhibitor of type I bone morphogenetic protein (BMP) receptors, and it has been widely used in research to explore the role of BMP signaling in various biological processes. Studies have reported that DMH1 inhibits cell growth in several cancer types and significantly reduces tumor progression when combined with anticancer drugs to overcome chemoresistance via BMP signaling pathway inhibition [16,17,18,19,20]. In this study, we have reported for the first time that DMH1 could effectively inhibit P-gp-mediated drug efflux, which may represent another potential mechanism to overcome chemoresistance.

## 2. Materials and Methods

### 2.1. Materials

The human castration-resistant prostate cancer (CRPC) cell line (PC-3) was obtained from the American Type Culture Collection (ATCC, Manassas, VA, USA). A docetaxel-resistant line (PC3-TxR) based on PC-3 was generously provided by Professor Moses S. S. Chow from the College of Pharmacy at Western University of Health Sciences (Pomona, CA, USA). Additionally, the docetaxel-resistant human leukemia cell line K562/Dox and daunorubicin (DNR) were kindly provided by Professor Ying Huang (College of Pharmacy, Western University of Health Sciences, Pomona, CA, USA). All cell lines were cultured in RPMI 1640 medium supplemented with 10% fetal bovine serum (FBS; Gibco) and 1% penicillin-streptomycin (GenClone^®^), under standard conditions of 5% CO_2_ at 37 °C. DMH1, (4-[6-[4-(1-Methylethoxy)phenyl]pyrazolo[1,5-a]pyrimidin-3-yl]-quinoline) (CAS# 1206711-16-1), was purchased from Selleck Chemicals LLC (Houston, TX, USA).

### 2.2. P-gp Activity and Inhibition

#### 2.2.1. DMH1 Cytotoxicity in Cell Lines with High and Low P-gp Expression

P-gp is overexpressed in PC3-TxR cells, while its expression is very low in PC3 cells [21]. To compare the difference in cytotoxicity of DHM1 on these two cell lines, PC3, PC3-TxR, and K562/Dox cells were seeded in a 96-well plate at a concentration of 8000 cells/well (*n* = 3). The cells were treated with DMH1 at different concentrations (0.1, 0.33, 1, 3.3, 10, 25, 50, 100, and 200 µM) for 72 h with DMSO (1%) as the negative control. The cell variability was then determined using a modified MTT Colorimetric method with CellTiter 96^®^ AQueous One Solution Cell Proliferation Assay (Promega, Madison, WI, USA). Briefly, 20 µL of CellTiter 96^®^ AQueous One Solution Reagent was added into each well, followed by incubation at 37 °C for 1 h. Absorbance at 490 nm was measured by a POLARstar spectrophotometer (BMG Labtech, Cary, NC, USA). The cell viabilities were normalized to control (DMSO 1%). The viability percentage is calculated using the following formula:

$$Viability(\%)=\frac{Absorbance(test)}{Absorbance\left(control\right)}\times 100$$

#### 2.2.2. Assessment of DMH1 Inhibition of P-gp Activity Using Calcein AM Retention Assay

The Vybrant Multidrug Resistance Assay Kit (Thermo Fisher Scientific, Ann Arbor, MI, USA) was used to evaluate multidrug resistance in prostate-resistant cancer cells PC3-TXR. Briefly, 50 μL of a series of dilutions of DMH1 or Verapamil hydrochloride, a calcium channel blocker that inhibits P-gp activity noncompetitively [22], was added to a 96-well microplate with PBS as a control. Then, about 5 × 10^5^ PC3-TXR cells in 100 μL RPMI1640 culture medium were added to each well. After incubation with DMH1 or Verapamil at 37 °C for 15 min, fluorescent substrate Calcein AM (50 μL) was added to the final concentration of 0.25 µM and incubated for another 15 min. The microplate was centrifuged at 200× *g* for 5 min. The supernatant was removed, and the cells were washed with cold RPMI 1640 medium twice. Calcein fluorescence was measured using a POLARstar spectrophotometer (BMG Labtech, Cary, NC, USA) at 494 nm excitation and 517 nm emission wavelengths. The results were expressed as the percentage of calcein-specific fluorescence retention in treated cells compared to untreated controls using the following equation:

$$\%intracellular\;calcein\;fluorescence=\frac{Fluorescence\;of\;treated\;cells}{fluorescence\;of\;untreated\;cells}\times 100$$

To visualize the intracellular retention of Calcein AM, the PC3 and PC3-TXR cells were seeded on coverslips and pre-treated with different concentrations of verapamil hydrochloride and DMH1 for 15 min. The cells were then incubated with 0.25 µM Calcein AM at 37 °C for 15 min. The cells were washed twice with PBS and fixed with 4% formaldehyde. Immunofluorescence images were taken with EVOS FL AMF4300 fluorescent imaging microscopy (Thermo Fisher Scientific, Waltham, MA, USA). Calcein AM fluorescence intensity was measured using the POLARstar spectrophotometer (BMG Labtech, Cary, NC, USA) at 494 nm excitation and 517 nm emission wavelengths.

### 2.3. Kinetic Evaluation of DMH1 Effects on P-gp-Mediated Efflux

#### 2.3.1. Inhibition of P-gp by DMH1 Enhancing Daunorubicin Accumulation

K562/Dox with P-gp overexpression was utilized to determine the inhibitory effect of DMH1 on P-gp [23]. The inhibition of P-gp-mediated drug efflux by DMH1 was assessed using a flow cytometry-based drug accumulation assay with DNR as the fluorescent marker. In this study, cyclosporin A (CsA), a common P-gp inhibitor, was used as the positive control. K562/Dox cells were cultured in RPMI 1640 containing 10% FBS and 1% penicillin/streptomycin at 37 °C. The cell suspension was collected by centrifuging at room temperature at 1500 rpm for 5 min. The cells were resuspended using fresh RPMI1640 medium and adjusted to a density of 1 × 10^4^ cells/mL. The cells were then incubated with DNR (10 µM) and various concentrations of DMH1 (0, 1, 3, 5, 10, and 20 μM) for 2 h. The cell pellets were collected by centrifugation and resuspended using cold PBS. The intracellular fluorescent intensity was measured using Attune NxT Acoustic Focusing Flow cytometry (Thermo Fisher Scientific, Waltham, MA, USA) with excitation at 488 nm and emission at 574/26 nm.

#### 2.3.2. Kinetic Analysis of P-gp Inhibition by DMH1

To evaluate the inhibition kinetics by DMH1 on P-glycoprotein (P-gp), the DNR efflux rate was measured in PC3-TxR cells. The cells were seeded into 96-well plates at a density of 1 × 10^4^ cells per well and incubated overnight at 37 °C. The next day, cells were pre-incubated for 30 min with either DMH1 or buffer control. This was followed by exposure to varying concentrations of DNR for 1.5 h. After washing with pre-warmed PBS, the cells were incubated in PBS containing either buffer or DMH1 for an additional 30 min to allow DNR efflux. The supernatant was then collected and transferred to black 96-well plates for fluorescence measurement. DNR fluorescence was recorded using a POLARstar spectrophotometer (BMG Labtech, Cary, NC, USA) with an excitation wavelength of 485 ± 12 nm and an emission wavelength of 590 ± 10 nm. Following fluorescence collection, cells were harvested for total protein quantification using the BCA assay (Thermo Fisher Scientific, Waltham, MA, USA). The data was analyzed under the assumption of Michaelis–Menten kinetics, and kinetic parameters, including maximum efflux rate (V_max_) and Michaelis constant (K_m_), were estimated via nonlinear regression using GraphPad Prism (version 10.4.1, GraphPad Software, Boston, MA, USA).

### 2.4. Mechanistic Study of DMH1 Inhibition on P-Glycoprotein

#### 2.4.1. P-gp ATPase Activity Assay

An ATPase assay using SB-MDR1 PREDEASYTM ATPase Kt (SOLVO Biotechnology, Budapest, Hungary) was utilized to evaluate how DMH1 influences P-gp ATPase activity. To determine vanadate-sensitive ATPase activity, 4 µg of SB-MDR1-Sf9 cell membrane fractions overexpressing ABCB1 (P-gp) was dispensed into a 96-well plate. Various concentrations of DMH1 were then added, both with and without sodium orthovanadate (Na_3_VO_4_). Additionally, a known P-gp activator, verapamil, was included in the inhibition test to examine whether DMH1 could suppress the maximum vanadate-sensitive ATPase response. Subsequently, 10 µL of MgATP solution (0.2 M) was introduced to each well except for those used to generate the phosphate standard curve. The mixture was incubated at 37 °C for 10 min, after which 100 µL of developer solution was added at room temperature. Following a 2 min incubation, 100 µL of blocker solution was introduced, and the plate was incubated for another 30 min at 37 °C. Finally, absorbance readings were taken at 590–630 nm using a POLARstar plate reader (BMG Labtech, Cary, NC, USA).

#### 2.4.2. Molecular Modeling-ABCB1

The molecular docking study was performed to investigate the interaction between the P-gp structure (PDB ID: 6QEX) and the small-molecule inhibitor DMH1 using the web-based docking tool CB-Dock2 (http://clab.labshare.cn/cb-dock, accessed on 22 January 2025). The crystal structure of P-gp (PDB ID: 6QEX) was retrieved from the Protein Data Bank and uploaded to CB-Dock2. The three-dimensional structure of DMH1 was obtained from PubChem (CID: 16720738) and converted into the appropriate format if necessary. The uploaded protein and ligand files were processed by CB-Dock2, which automatically identified potential binding pockets based on a curvature-based algorithm. The docking software calculated the center and size of the identified cavities and performed blind docking. The optimal docking conformation was selected based on the lowest predicted binding energy and the presence of favorable interactions, such as hydrogen bonds, hydrophobic interactions, and π-π stacking.

### 2.5. Statistics

All data are presented as means ± SEM (standard error of mean) unless otherwise indicated in the figures. Comparisons of the means between two groups were performed using Student’s *t* test, while one-way ANOVA was used for the comparisons among multiple groups, followed by the post-hoc Bonferroni test. A *p*-value < 0.05 was considered statistically significant. All the tests were conducted using GraphPad Prism 8.3.1 (version 10.4.1, GraphPad Software, Boston, MA, USA).

## 3. Results

### 3.1. DMH1 Exhibits No Cytotoxicity in P-gp-Overexpressing or -Deficient Cells

**Figure 1:**
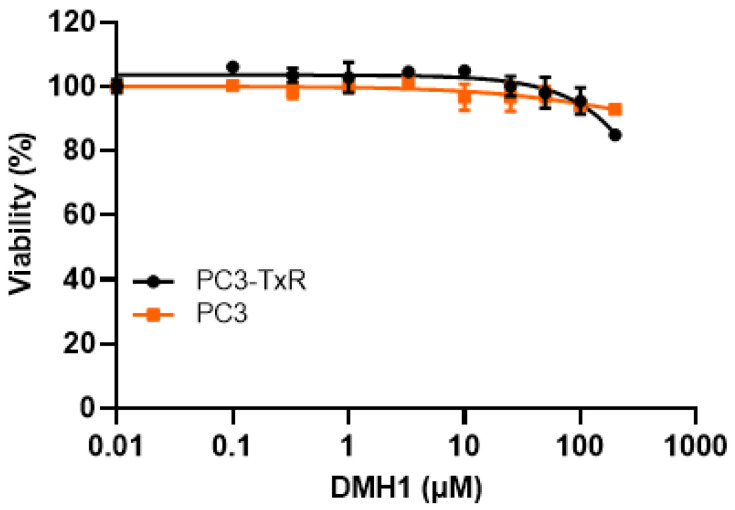
**Cytotoxicity of DMH1 in sensitive and chemoresistant prostate cancer cells.** The cytotoxicity of DMH1 was determined by the modified MTT colorimetric assay. The cells were treated with vehicle DMSO, or DMH1 at concentrations from 0.1 µM to 200 µM for 72 h. The control viability (DMSO) was designated as 100%, and the cell viabilities were normalized to the control cell viability. DMH1 shows no cytotoxicity to both chemosensitive PC3 and chemoresistant PC3-TXR cells, even at the very high concentration of 200 µM. Additionally, there is no significant difference in the cytotoxicity of DMH1 between sensitive and resistant cells.

Our RT-PCR confirmed that P-gp is overexpressed in PC3-TxR cells but not in PC3 cells, which is consistent with previous reports [23,24] (Figure 1). Cytotoxicity assays demonstrated that DMH1 does not induce statistically significant cytotoxic effects in either PC3 or PC3-TxR cells across a broad concentration range (0.1 µM to 100 µM). Even at 200 µM, cell viability remained high—92.9% in PC3 cells and 84.9% in PC3-TxR cells (Figure 1). Typically, P-gp substrates exhibit differential cytotoxicity between P-gp-deficient and P-gp-overexpressing cells due to active efflux. However, the comparable cytotoxic profiles observed in both PC3 and PC3-TxR cells suggest that DMH1 is not a substrate of P-gp.

### 3.2. DMH1 Effectively Inhibits P-gp Efflux Function in Calcein AM Assays

Although DMH1 is not a P-gp substrate, its ability to modulate P-gp function remains unclear. To investigate whether DMH1 can inhibit P-gp-mediated efflux and thereby enhance intracellular drug accumulation, we performed a series of Calcein AM uptake assays. Calcein AM is a well-characterized P-gp substrate that passively diffuses across the plasma membrane and is cleaved by intracellular esterases into fluorescent calcein, which accumulates in the cytoplasm.

**Figure 2:**
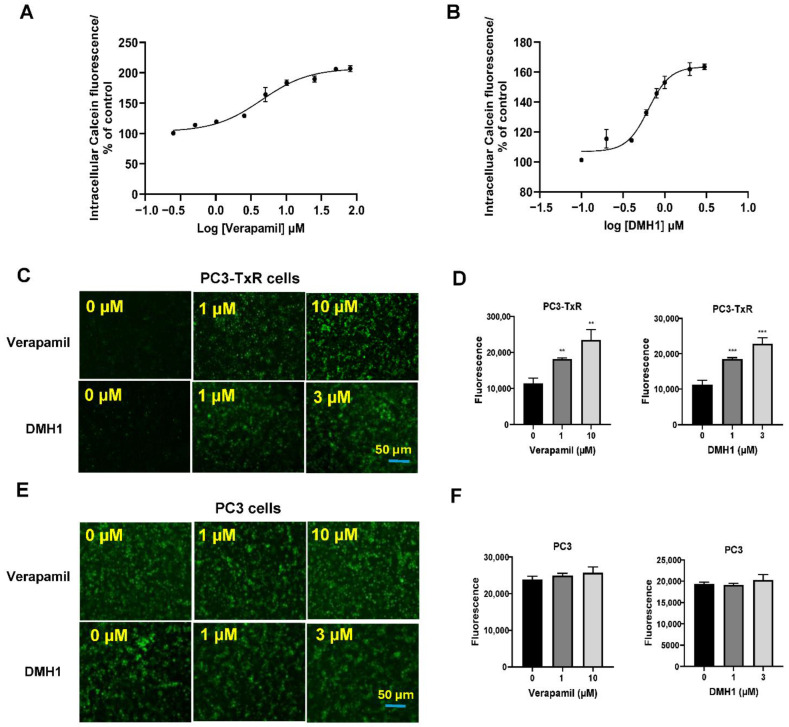
**DMH1 inhibits P-gp1 efflux activity for Calcein AM in chemoresistant prostate cancer cells.** The Calcein-AM assay was performed using the Vybrant Multidrug Resistance kit (Thermo Fisher Scientific, Ann Arbor, MI, USA). The intensity of intracellular calcein fluorescence markedly increased with rising concentrations of P-gp inhibitor verapamil (**A**) and DMH1 (**B**) in PC3-TxR cells. In consistent DMH1, like P-gp inhibitor Verapamil, increased intracellular accumulation of calcein-AM in a dose-dependent manner in the chemoresistant PC3-TxR cells, which overexpress P-gp1 protein as determined by image analysis (**C**) and microplate spectrofluorometer analysis (**D**). However, in the chemosensitive PC3 cells, DMH1, similar to verapamil, did not exert a significant effect on Calcein AM accumulation (**E**,**F**) (** *p* < 0.01, *** *p* <0.001).

In the P-gp-overexpressing PC3-TxR cells, P-gp leads to increased extrusion of Calcein AM before hydrolysis occurs, reducing intracellular fluorescence. The Vybrant Multidrug Resistance assay demonstrated that intracellular calcein fluorescence intensity markedly increased with rising concentrations of verapamil (a known P-gp inhibitor for positive control) and DMH1 in P-gp-overexpressing PC3-TxR cells, suggesting that DMH1 effectively inhibits P-gp efflux function (Figure 2A,B). In consistence, DMH1 and verapamil significantly increased intracellular accumulation of Calcein AM in a dose-dependent manner in the P-gp-overexpressing PC3-TxR cells, respectively, as determined by both image analysis and microplate spectrofluorometer analysis (Figure 2C,D). In contrast, neither DMH1 nor verapamil had a noticeable effect on Calcein AM accumulation in P-gp-lacking PC3 cells (Figure 2E,F). Taken together, these results indicate that DMH1 effectively inhibits P-gp efflux function.

### 3.3. DMH1 Inhibits P-gp-Mediated Drug Efflux to Enhance Intracellular Accumulation of Daunorubicin

**Figure 3:**
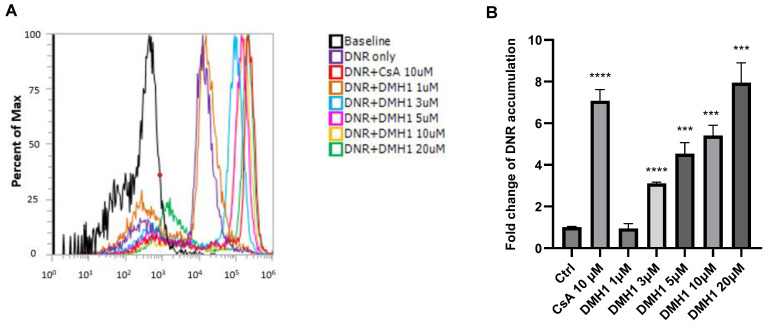
**Inhibition of P-gp by DMH1.** (**A**) Flow cytometric histograms showing intracellular daunorubicin (DNR) fluorescence in K562/DOX cells indicate that both the positive control cyclosporine A (CsA) and DMH1 increased DNR accumulation. (**B**). The concentration-dependent effect of DMH1 or CsA on P-gp substrate DNR accumulation in K562/Dox cells. The positive control, 10 µM CsA, inhibited DNR efflux, leading to a high intracellular concentration. DMH1 also significantly inhibited P-gp activity in a dose-dependent manner. (*** *p* < 0.001, **** *p* < 0.0001 compared to the Ctrl group).

The K562/DOX cell line, which exhibits doxorubicin resistance due to P-gp overexpression, was used to assess the inhibitory effect of DMH1 on P-gp activity as well [25]. No significant cytotoxic effects were observed in this cell line following DMH1 treatment (Supplementary Figure S1). Flow cytometry was performed to evaluate the inhibition of DNR efflux in K562/DOX cells following treatment with 10 µM cyclosporin A (a known P-gp inhibitor as a positive control) or DMH1 at concentrations ranging from 1 µM to 20 µM. Flow cytometric histograms indicate that both cyclosporin A and DMH1 increased intracellular DNR accumulation (Figure 3A). As quantified in Figure 3B, 10 µM cyclosporin A treatment resulted in a 7.1-fold increase in intracellular DNR concentration compared to basal control. Similarly, DMH1 significantly inhibited P-gp activity in a dose-dependent manner and increased intracellular DNR concentrations by 3.1-fold at 3 µM, 4.5-fold at 5 µM, 5.4-fold at 10 µM, and 7.9-fold at 20 µM relative to the basal control (Figure 3B). These findings further support that DMH1 effectively inhibits P-gp efflux function, thereby enhancing intracellular retention of chemotherapeutic agent DNR in P-gp-overexpressing cells.

### 3.4. DMH1 Is a Noncompetitive Inhibitor of P-gp

**Figure 4:**
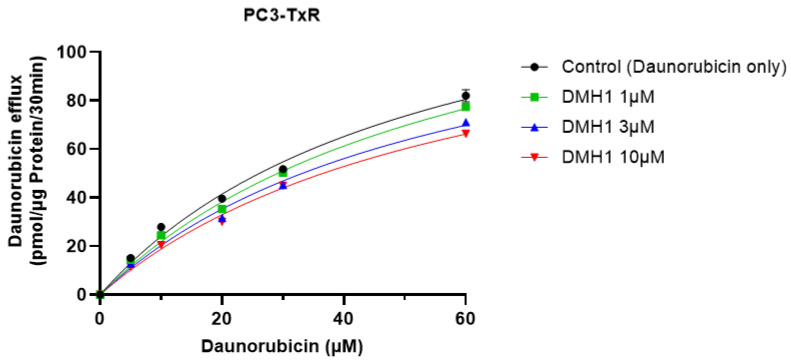
**DMH1-mediated enzyme kinetic mechanism underlying the P-gp-mediated efflux of daunorubicin in PC3-TxR cells.** The cells were pre-treated with buffer alone or DMH1, followed by treatment with various concentrations of daunorubicin. After washing and incubating with PBS containing either buffer alone or DMH1, the fluorescence in the supernatant was measured. The curve indicates that the concentration-dependent effects of DMH1 on the efflux of daunorubicin followed Michaelis–Menten kinetics.

To determine the inhibitory mechanism of DMH1 on P-gp activity, a DNR efflux assay was conducted in P-gp-overexpressing PC3-TxR cells. Cells were pre-treated with DMH1 (3 µM or 10 µM) prior to exposure to varying concentrations of DNR. Following incubation and washing, the fluorescence intensity in the supernatant was measured to quantify DNR efflux. Kinetic parameters were derived using Michaelis–Menten analysis. As shown in Table 1 and Figure 4, DMH1 at 3 µM and 10 µM significantly reduced the maximal efflux rate (V_max_) of DNR, while the substrate affinity (K_m_) of P-gp remained unchanged. Such inhibition typically arises when an inhibitor binds to an allosteric site distinct from the substrate-binding pocket, thereby reducing the transport capacity (V_max_) without affecting substrate affinity (K_m_). Therefore, DMH1 functions as a noncompetitive inhibitor of P-gp by interacting with an allosteric site distinct from the substrate-binding sites.

### 3.5. DMH1 Inhibits P-gp ATPase Activity Likely via Allosteric Interaction or Conformational Modulation

**Figure 5:**
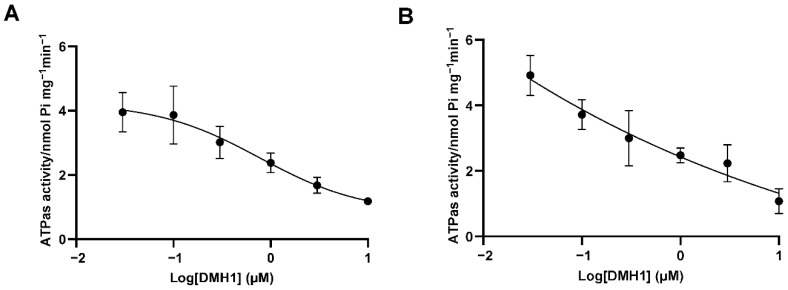
**Effects of DMH1 on the ATPase activity of human P-gp.** An SB-MDR1 PREDEASYTM ATPase Kit (SOLVO Biotechnology, Budapest, Hungary) with the cell membrane overexpressing ABCB1 was used to assess the effects of DMH1 on the activity of P-gp ATPase. (**A**) DMH1 at various concentrations from 0.03 µM to 10 µM could inhibit vanadate-sensitive ATPase activity in a dose-dependent manner. (**B**) DMH1 at various concentrations from 0.03 µM to 10 µM could also inhibit verapamil-induced ATPase activity in a dose-dependent manner.

Given that DMH1 functions as a noncompetitive inhibitor of P-gp, we next examined its effect on ATPase activity, which is crucial for ATP hydrolysis and fuels P-gp-mediated drug transport. A P-gp ATPase assay was conducted using the SB-MDR1 PREDEASY™ ATPase Kit with membrane preparations overexpressing P-gp. DMH1 was tested at concentrations ranging from 0.03 µM to 10 µM. Since P-gp ATPase activity is sensitive to sodium orthovanadate (Na_3_VO_4_), which inhibits ATP hydrolysis catalyzed by ABC transporters, we quantified the vanadate-sensitive fraction to isolate specific P-gp-associated activity from background ATPase activity. As shown in Figure 5A, DMH1 inhibited the vanadate-sensitive ATPase activity in a dose-dependent manner, suggesting that it may interfere with ATP hydrolysis either by binding to an allosteric site or by inducing conformational changes that impair catalytic efficiency.

In contrast, verapamil, a well-characterized competitive P-gp inhibitor and substrate, interacts directly with the substrate-binding site, exhibiting a biphasic effect on ATPase activity [26]. At low concentrations, verapamil stimulates ATPase activity, enhancing efflux, whereas at high concentrations, it inhibits ATPase activity by stabilizing the P-gp-substrate complex, thereby reducing ATP hydrolysis and transport. Unlike verapamil, DMH1 does not stimulate ATPase activity at any concentration tested (Figure 5B). Instead, it consistently inhibits ATPase activity, likely by binding to an allosteric site or altering the conformational dynamics of P-gp.

### 3.6. Molecular Docking Reveals That DMH1 Binds to an Allosteric Site of P-gp

**Figure 6:**
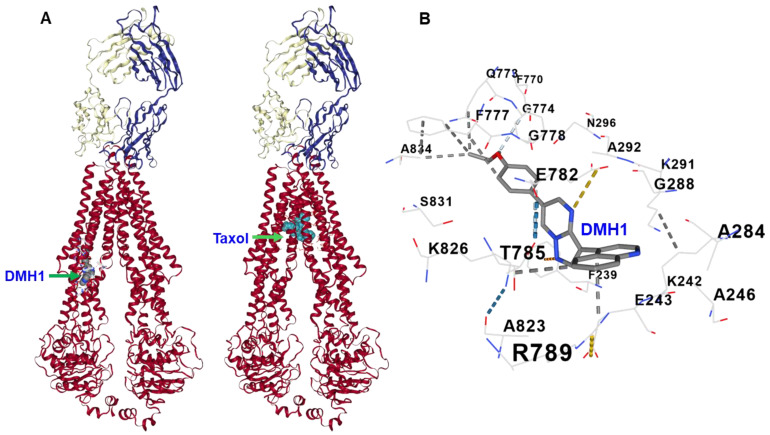
**Molecular docking analysis shows that DMH1 binds to the allosteric site of human P-gp, distinct from both the substrate-binding cavity and the NBD of P-gp.** (**A**) Structural representation of the DMH1 binding site on human P-gp (PDB ID: 6QEX). DMH1 is docked within a distinct allosteric pocket located in the TMD, spatially separated from both the substrate Taxol-binding cavity and the NBDs. (**B**) Detailed view of molecular interactions between DMH1 and residues within the allosteric pocket. DMH1 forms hydrophobic interactions with Phe 728, Leu 975, and Val 978, and establishes hydrogen bonds with Gln 721 and Asn 822. These interactions may contribute to the stabilization of DMH1 within the pocket and influence P-gp activity through allosteric modulation.

Further molecular docking analysis using the human P-gp crystal structure (PDB ID: 6QEX) revealed that DMH1 binds to an allosteric site within the TMD of P-gp, distinct from both the substrate-binding cavity (e.g., the Taxol site) and the NBD (Figure 6A). This allosteric pocket appears to be positioned to influence the conformational flexibility of P-gp, which is crucial for its transport function. Within this site, DMH1 establishes stabilizing interactions with several key amino acid residues, including hydrophobic contacts with Phe 728, Leu 975, and Val 978, as well as hydrogen bonding with Gln 721 and Asn 822 (Figure 6B). Additionally, electrostatic interactions with Glu 875 may further enhance its binding stability, anchoring DMH1 in a region that could disrupt the dynamic coupling between the TMDs and NBDs. These findings further support that DMH1 acts as a noncompetitive inhibitor by targeting an allosteric regulatory site of P-gp, consistent with our experimental data.

## 4. Discussion and Conclusions

Inhibiting P-gp activity to increase intracellular drug accumulation is a critical approach to overcoming drug resistance and improving therapeutic outcomes. Efforts to develop P-gp inhibitors have led to several drug candidates, including verapamil, cyclosporine A, laniquidar, valspodar, and various natural and modified compounds [1,9,10,11,12]. They act through multiple mechanisms for P-gp activity inhibition, such as competitive inhibition directly binding to P-gp’s substrate-binding sites and non-competitive/allosteric inhibition by binding to a site distinct from the substrate-binding sites without competing with substrates and other pathways [1,27,28,29,30]. Nevertheless, all these candidates have not achieved clinical success due to challenges such as poor selectivity, toxicity, and limited efficacy [13,14,15]. Consequently, there is a growing need for novel P-gp inhibitors that offer improved safety, specificity, and potency.

In this study, we have demonstrated that DMH1 exhibited no cytotoxicity in P-gp overexpressing or deficient cells, while it effectively inhibited P-gp efflux in Calcein AM assays and increased intracellular DNR concentrations without affecting its binding affinity. All these findings support that DMH1 is not a competitive P-gp inhibitor and does not compete directly with DNR at the P-gp binding site. Instead, DMH1 likely modulates P-gp efflux activity through an allosteric mechanism. Consistent with this, efflux kinetics analysis reveals that DMH1 noncompetitively inhibits DNR efflux and raises intracellular drug concentrations without altering the binding affinity.

A possible mechanism of the inhibitory activity of DMH1 is the suppression of P-gp ATPase activity, which is essential for P-gp-mediated drug efflux. Our P-gp ATPase assay demonstrated that DMH1 inhibited ATPase activity in a dose-dependent manner. Since P-gp substrates do not typically interfere with the ATPase catalytic cycle, this further supports DMH1’s role as an allosteric modulator. Moreover, DMH1 inhibited verapamil-stimulated maximal vanadate-sensitive ATPase activity, with ATPase activity inversely correlated with DMH1 concentration. These results suggest that DMH1 may interfere with the ATP hydrolysis process or prevent verapamil from stimulating ATP hydrolysis.

Furthermore, we performed docking studies of DMH1 with the P-gp crystal structure (PDB ID: 6QEX). Our structural analysis demonstrated that DMH1 binds to an allosteric site within the TMD, distinct from both the primary substrate-binding cavity and NBDs. Within this site, DMH1 forms stabilizing interactions with Phe 728, Leu 975, and Val 978 through hydrophobic contacts, while it also engages in hydrogen bonding with Gln 721 and Asn 822. Additionally, electrostatic interactions with Glu 875 may further strengthen its positioning within the allosteric site. By binding in this region, DMH1 likely stabilizes an inward-facing conformation of P-gp, thereby disrupting the conformational transitions necessary for substrate extrusion. This structural restriction may prevent P-gp from transitioning to its outward-facing state, effectively reducing its efflux capacity. Furthermore, its interaction within the TMD may disrupt allosteric communication between the TMDs and NBDs, impairing ATP hydrolysis efficiency. Since ATP hydrolysis is essential for substrate transport, such interference could significantly weaken the efflux function of P-gp, reducing its ability to pump out chemotherapeutic agents. This mechanistic insight aligns with our experimental ATPase assay results, further supporting that DMH1 acts as an allosteric modulator rather than a direct competitive inhibitor. Unlike traditional competitive inhibitors that directly block substrate binding, allosteric modulators like DMH1 offer a distinct mechanism of P-gp inhibition that may lower the risk of resistance commonly associated with direct P-gp inhibitors. Consequently, our findings suggest that DMH1 may be used as a molecular probe to study P-gp-mediated drug efflux mechanisms, as it has been newly identified as a P-gp inhibitor. Moreover, DMH1 may represent a promising lead compound for further evaluation of its safety and efficacy in overcoming multidrug resistance and enhancing the therapeutic effectiveness of chemotherapeutic agents in vivo. Cytotoxicity assays demonstrated that DMH1 had low toxicity in vitro, and it was also well tolerated at a dose of 5 mg/kg in mice [19]. Nevertheless, comprehensive toxicological evaluations, particularly chronic toxicity studies, along with detailed pharmacokinetic properties and possible drug–drug interactions, are essential to assess its clinical therapeutic applications.

Despite these encouraging results, several limitations must be addressed in future research. This study primarily utilized in vitro models, which may not fully capture the complexities of drug interactions and resistance mechanisms in vivo. The cellular responses observed in controlled laboratory conditions could differ significantly in a more dynamic biological environment. In addition, while our CB-Dock docking results suggest that DMH1 may bind to an allosteric site, which is consistent with our biochemical data supporting noncompetitive inhibition, additional validation is essential. More advanced computational methods (e.g., molecular dynamics simulations and MM/GBSA free energy calculations) or experimental structural approaches (e.g., cryo-EM, X-ray crystallography) are needed to substantiate this model and more accurately define the binding mechanism of DMH1. This is particularly important because accurately predicting ligand binding to P-glycoprotein (P-gp) is inherently challenging due to its highly dynamic nature, which involves substantial conformational rearrangements during substrate binding and transport. This complexity is further compounded by P-gp’s polyspecificity for structurally diverse small molecules. Finally, while our findings offer valuable insight into DMH1’s inhibitory potential, the precise molecular mechanisms underlying its action remain to be fully elucidated. Future investigation is needed to clarify the exact binding dynamics between DMH1 and P-gp, leveraging high-resolution structural analyses and advanced biochemical assays to further validate its mechanism of action.

## Figures and Tables

**Table 1:** Kinetic parameters of P-gp mediated effluxes of daunorubicin in PC3-TxR cells treated with DMH1.

	V_max_ (pmol/µg Protein/30 min)	K_m_ (µM)
Daunorubicin only	180.10 ± 14.00	59.16 ± 7.44
+1 µM DMH1	154.78 ± 3.03	61.03 ± 2.14
+3 µM DMH1	135.66 ± 6.87 *	59.47 ± 4.72
+10 µM DMH1	132.64 ± 2.34 **	59.95 ± 2.95

V_max_ represents the maximal efflux rate of daunorubicin. K_m_ is the Michaelis–Menten constant. * indicates comparison with control (Daunorubicin only), * *p* < 0.05, ** *p* < 0.01. Data are presented as mean ± SEM.

## Data Availability

All data and reagents are available from the authors upon request.

## Supplementary Materials

The following supporting information can be downloaded at https://www.mdpi.com/article/10.3390/biomedicines13081798/s1: Supplementary Figure S1: Cytotoxicity of DMH1 in K562/Dox cells. The cytotoxicity of DMH1 was determined by the modified MTT colorimetric assay. The cells were treated with vehicle DMSO, or DMH1 at concentrations from 0.1 µM to 200 µM for 72 h. No significant cytotoxic effects were observed in this cell line following DMH1 treatment, even at the very high concentration of 200 µM.
