# Supplementary material for: Allosteric Inhibition of P-Glycoprotein-Mediated Efflux by DMH1
Source: Biomedicines. 2025 Jul 23;13(8):1798. doi: 10.3390/biomedicines13081798 (PMC12383532; doi:10.3390/biomedicines13081798)

Supplementary Figure S1: Cytotoxicity of DMH1 in K562/Dox cells. The cytotoxicity of DMH1 was determined by the modified MTT colorimetric assay. The cells were treated with vehicle DMSO, or DMH1 at concentrations from 0.1 µM to 200 µM for 72 h. No significant cytotoxic effects were observed in this cell line following DMH1 treatment, even at the very high concentration of 200 µM.

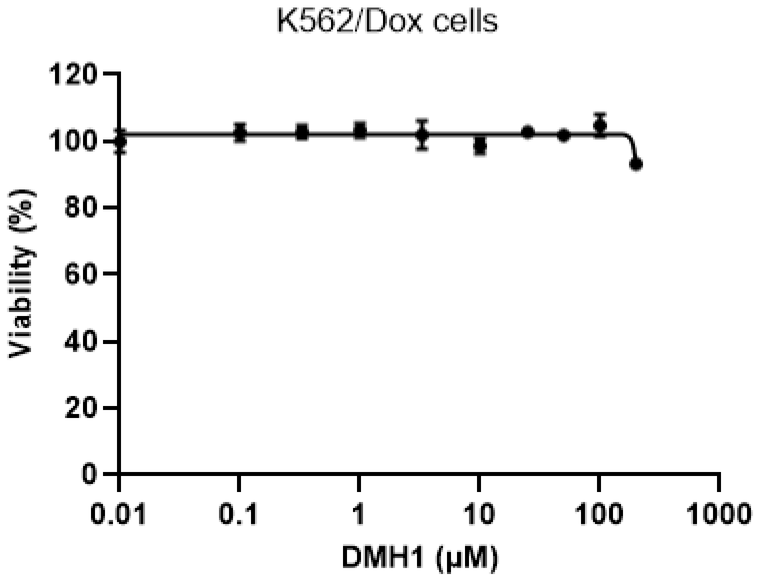

Supplement: Supplementary file 1 [file biomedicines-13-01798-s001.zip › biomedicines-3750914-supplementary.pdf]
